# Nanoparticles Modified with Cell-Penetrating Peptides: Conjugation Mechanisms, Physicochemical Properties, and Application in Cancer Diagnosis and Therapy

**DOI:** 10.3390/ijms21072536

**Published:** 2020-04-06

**Authors:** Isabel Gessner, Ines Neundorf

**Affiliations:** 1 Department of Chemistry, Inorganic Chemistry, University of Cologne, Greinstr 6, 50939 Cologne, Germany; isabel.gessner@uni-koeln.de; 2 Department of Chemistry, Biochemistry, University of Cologne, Zuelpicher Str. 47a, 50674 Cologne, Germany

**Keywords:** cell-penetrating peptides, nanoparticles, cancer medicine, conjugation mechanism

## Abstract

Based on their tunable physicochemical properties and the possibility of producing cell-specific platforms through surface modification with functional biomolecules, nanoparticles (NPs) represent highly promising tools for biomedical applications. To improve their potential under physiological conditions and to enhance their cellular uptake, combinations with cell-penetrating peptides (CPPs) represent a valuable strategy. CPPs are often cationic peptide sequences that are able to translocate across biological membranes and to carry attached cargos inside cells and have thus been recognized as versatile tools for drug delivery. Nevertheless, the conjugation of CPP to NP surfaces is dependent on many properties from both individual components, and further insight into this complex interplay is needed to allow for the fabrication of highly stable but functional vectors. Since CPPs per se are nonselective and enter nearly all cells likewise, additional decoration of NPs with homing devices, such as tumor-homing peptides, enables the design of multifunctional platforms for the targeted delivery of chemotherapeutic drugs. In this review, we have updated the recent advances in the field of CPP-NPs, focusing on synthesis strategies, elucidating the influence of different physicochemical properties, as well as their application in cancer research.

## 1. Introduction

Safe and efficient drug delivery is still one of the main bottlenecks in pharmaceutical drug development. The plasma membrane represents an effective barrier to all large and charged molecules, and thus, particularly for those compounds, the design of carriers that support transport inside cells is one of the key challenges. Cell-penetrating peptides (CPPs) have emerged as versatile tools to facilitate drug delivery. These often-small peptide sequences are coupled to a biologically active cargo and are able to transport it across the plasma membrane. On the other side, nanoparticles (NPs) provide a great opportunity in biomedical sciences and have been widely used to increase the pharmacokinetic properties of bioactive drugs. During the last years, CPPs have been a welcomed tool to combine favorable properties of both compound classes. One major focus is to increase cell penetration and delivery to intracellular target destinations of NPs by decorating them with CPPs. Therefore, during the last years, a variety of NP-CPP conjugates has been developed, as demonstrated in a number of excellent reviews, which mainly focus on their application in the field of biomedicine [1,2,3,4].

Nevertheless, the interplay between CPPs and NPs is highly complex, and the choice of the conjugation method plays a pivotal role, leading to highly efficient intracellular vectors or in contrast to impairment of peptide functionality. Based on our experience, we believe that understanding this chemical interaction, the stability of formed bonds and their influence on potential changes in physicochemical properties of CPPs and NPs are essential to developing conjugates that possess high cell internalization efficiencies and biocompatibilities, requisites that are needed for their successful translation into clinics. Therefore, within this current work, we attempted to highlight recent progress made in the employed conjugation mechanisms of CPP-NP conjugates, discussing their advantages and disadvantages and potentially useful techniques for the characterization of resulting physicochemical properties. Moreover, we have updated the field of biomedical applications of CPP-NP conjugates in cancer medicine with approaches that were performed during the last two years to depict the current state of the art.

## 2. Cell-Penetrating Peptides

Since their discovery in 1988 [5,6], cell-penetrating peptides (first referred to as “protein transduction domains, PTDs”) have been used in many studies as effective non-viral delivery systems. CPPs are usually small in size (5–30 amino acids) and arrest attention by their nearly non-toxic and non-immunogenic behavior. Many of such peptide sequences identified so far have been used as versatile vehicles for the intracellular transport of various cargos, such as small molecule drugs, peptides, proteins, nucleic acids, or particles. In spite of this, it was not the intention of this review to exhaustively summarize all facets of CPP research, but more to highlight the main key facts about their classification, ways of function, and usage in biomedical research, with the focus on their application within CPP-NP constructs. For detailed information about CPPs, the readers are referred to other recent reviews [7,8,9].

### 2.1. Types

CPPs can be grouped in different ways, e.g., based on their type of origin or physicochemical properties. The latter one is closely related to their mechanism of cellular uptake and will, therefore, be considered. In this context, CPPs are divided into three classes depending on their amino acid sequences, namely, cationic, amphipathic, and hydrophobic, while the group of cationic peptides is the largest one [8]. Cationic CPPs are characterized by high contents of basic amino acids, such as lysine, ornithine, arginine, and histidine. No doubt, one of the most prominent sequences is that derived from the transactivator protein (Tat) of human deficiency virus type 1 (HIV), e.g., Tat(48–60) [5] (Table 1). But also poly-arginine peptide sequences (R_5_–R_9_) fall into this group and are frequently used, particularly for the transport of proteins or nucleic acids [10]. Many of the amphipathic CPPs form secondary structures, alpha helices, or beta-sheets when in contact with lipid membranes, facilitating lipid interaction and membrane perturbation [11]. One example appeared in our group, namely, sC18, which comprises the *C*-terminal sequence of the cationic antimicrobial protein CAP18 that belongs to the family of cathelicidins [12]. Other examples of this group are represented by the peptides VP22, penetratin, or model amphipathic peptide (MAP) [13,14,15]. The last group is formed by hydrophobic CPPs, with sequences recently identified by orthogonal high-throughput screening [16].

### 2.2. Uptake Mechanisms of CPP

CPPs usually enter cells in different ways roughly divided into energy-independent direct penetration or energy-dependent endocytotic processes. Direct penetration generally takes place, when higher concentrations of CPPs are applied, and follows various mechanisms involving membrane interaction and disturbance [17]. Notably, the theoretical models explaining the energy-independent entry of CPPs are strongly based on models highlighted for the membrane interaction of antimicrobial peptides (AMPs) and include the ‘barrel-stave’ or ‘toroidal-pore’ pathway, the ‘carpet’ model, inverted micelle formation, as well as membrane thinning processes [17,18]. On the other hand, endocytosis is mainly observed when larger cargos are attached to the CPP and directly implicates one major drawback in CPP usage. After endosomal uptake, the CPP-cargo conjugate has to be released out of the vesicles to reach the defined site of action. To increase the probability of endosomal release, CPPs can be modified in different ways; for example, by attaching fusogenic peptide sequences. These are usually derived from viral fusion proteins that, depending on the acidic environment inside the endosomes, undergo structural rearrangements, leading to the formation of alpha-helices that disturb and destabilize the vesicle membrane and promote the escape of the CPP or CPP-cargo construct after endosome disruption [12,19]. Another strategy is to co-administer small weak bases, which increase the pH of endosomes by protonation and cause osmotic swelling and rupture of the vesicles by the so-called proton sponge effect. Following this mediates endosomal swelling and disruption with the final release of the entrapped cargo [20]. For instance, very prominent is the use of chloroquine, but recently other compounds have also been developed in this context [21].

However, for both major processes mentioned (endocytosis or direct penetration), it is assumed that positively charged CPPs, like penetratin, Tat, sC18, VP22, and the poly-arginines, are first attracted by negatively charged groups at the outer membrane surface, which induces binding and aggregation, and subsequent induction of the distinct entry mechanisms. Importantly, arginine and lysine residues play major roles for this synergy by forming bi- or monodentate hydrogen bonds, respectively, to phosphate, sulfate, or carboxylate groups. For instance, particularly proteoglycans have been identified to be important, and thus, highly cationic CPPs like Tat and poly-arginines are significantly less transduced in cells deficient in heparan sulfate [22,23]. On the other side, for amphipathic CPPs, like penetratin, the interaction with the lipid core components in the membrane is of importance, too, and often supports conformational transitions that ease the creation of transient membrane pores [17].

Overall, the cellular uptake of CPPs is more than a complex process, which is still not fully elucidated and highly debated. One of the challenges in CPP uptake studies is the many factors that have to be considered for every CPP sequence, such as cell type, cargo size, temperature, and concentration used, making it still difficult to draw a conclusive and general picture. Nevertheless, in our opinion, it is of significant importance to evaluate and gain knowledge about the entry mechanism of a given CPP formulation for understanding and modulating the expected biological response.

### 2.3. Application

Since their first report, CPPs have been used in various applications in vitro and in vivo. First, they were mainly applied to enhance cell permeation of proteins, and that is why they initially were entitled protein transduction domains, PTDs. Meanwhile, the portfolio of successfully delivered biologically active compounds has steadily increased and, besides peptides and proteins, strives diverse other compound classes, such as nucleic acids (pDNA, siRNA, aptamers, as well as peptide nucleic acids PNAs, etc.), small molecules (organic or, e.g., inorganic complexes), and particles of various sizes [24]. Thereby, the cargos are attached by different means, including non-covalent and covalent methods, whereas small molecules are in most of the cases covalently bound to the CPP sequences [25]. Generally, the attached cargos have manifold functions, and their medical application is often found in the fields of diagnosis (often imaging) and therapeutic applications. Although the amount of CPP sequences reported in the literature is steadily increasing, still there is no CPP-based treatment that has been approved by the FDA. This is mainly due to a lack of proteolytic stability, selectivity, and the problem of efficient endosomal release. However, many CPPs are in (pre)clinical studies, showing promising results in treating cancers and other diseases, and let hope to continue for their success in the future [26]. To increase the probability of obtaining more effective and safer biomedicals, a combination of CPPs with NPs might be a favored strategy, which has been discussed below.

## 3. Nanoparticles

**Figure 1:**
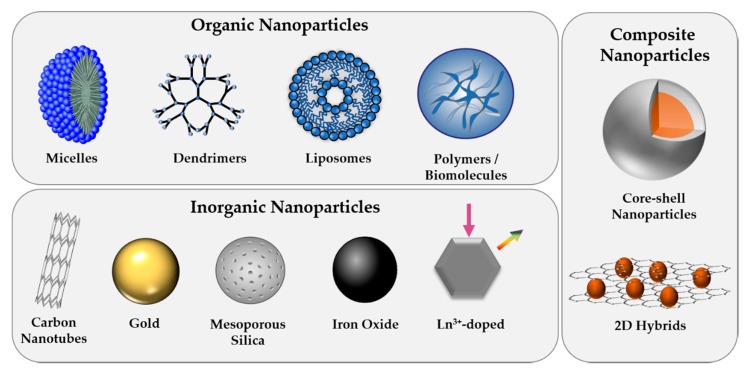
Overview of commonly used nanoparticle (NP) types, classified as organic, inorganic, or composite structures.

Increasing know-how and continuous progress in engineering materials at the nanoscale with tunable physicochemical properties has led to the indispensable implementation of NPs in various fields of our everyday life, including the medical sector. With the potential to revolutionize our current treatment and visualization of various diseases, including cancers, NPs have not only been developed for biosensing and bioimaging to allow for early detection of dysfunctional cellular pathways but also for therapeutic purposes [27,28]. The controlled killing of diseased cells is commonly performed through the application of heat (photothermal therapy, hyperthermia), ultrasound, radiation, activation of reactive oxygen species (photodynamic therapy), gene and immune-modulation, the delivery of high payloads of drug molecules, or combinations thereof [29]. Based on their high active surface area compared to their small volume, NPs do not only serve as excellent carriers for therapeutic (bio)molecules but, depending on the composition, often possess inherent features that allow for their intracellular tracking or therapeutic use. Engineered NPs that have been designed for biomedical applications comprise a broad range of material classes, including organic, inorganic, and more complex composite structures (Figure 1) [30]. This chapter is intended to give a brief overview of the most commonly employed NP types and to highlight their outstanding characteristics.

### 3.1. Organic

Organic NPs that comprise polymeric as well as micellar, liposomal, and highly branched dendrimeric structures have become increasingly important in biomedicine, especially for drug delivery applications [31]. Among a vast variety of polymer-based nanostructures that have been employed for medicinal purposes, nonbiodegradable polymers, such as polystyrene or poly(methyl methacrylate) (PMMA), have demonstrated inflammatory reactions, as well as chronic toxicity [32]. Therefore, nowadays, the leading choices have shifted to more biocompatible and biodegradable alternatives, such as poly(lactic acid) (PLA), poly(lactic-co-glycolic) acid (PLGA), and polysugars like chitosans or dextrans [33]. With regard to the protected delivery of therapeutic molecules, liposomes that resemble cellular bilayers have emerged as highly efficient carrier systems as they possess hydrophobic and hydrophilic sub-structures that allow for the encapsulation of multiple drug types. The tunable physicochemical properties, which rely mainly on the choice of lipid and the preparation method, along with their high biocompatibility has led to the first FDA approved nano-drug in 1995, Doxil^®^, doxorubicin-loaded liposome [34]. In order to provide specific interaction with cells, organic NPs are often functionalized with biomolecules. Alternatively, self-assembly of natural biomolecular building blocks has been used for the formation of organic NPs that are made up of proteins, oligonucleotides, and peptides, including CPPs [35].

### 3.2. Inorganic NPs

Compared to their organic counterparts, inorganic NPs offer high mechanical strength and chemical stability in the biological milieu and are thus very resistant to enzymatic degradation and disintegration through biological fluids. Moreover, their fabrication with high control over obtained size, morphology, and composition allows for the tailored design of sensitive nanoplatforms with desired electrical, optical, and magnetic properties needed for their use in specific biomedical applications [36]. Given their simple synthesis along with their tunable size, morphology, and porosity, silica NPs have become predominant among all inorganic carrier materials for drug delivery applications. The efficient entrapment of high payloads of cargo molecules within their porous structure, followed by the controlled and sustained release of loaded molecules upon endogenous or exogenous stimuli, such as light, temperature, ultrasound, or pH, has made mesoporous silica nanostructures highly promising delivery vehicles [37]. Moreover, while inorganic NPs are often depicted as less biocompatible compared to lipid or polymer-derived particles, the degradation mechanism of mesoporous silica particles has been studied in detail and revealed silicic acid as biocompatible and clearable degradation product [38].

Likewise, iron oxide NPs have received a special status among inorganic carriers when it comes to toxicity concerns as iron constitutes an essential element in our body, and iron ions released by iron oxide nanostructures have been shown to be reincorporated into the natural cell metabolism. In fact, ferumoxytol, a carbohydrate-coated iron oxide NP, is an FDA approved agent used for the treatment of iron deficiencies [39]. The superparamagnetic behavior and catalytic activity of iron oxide NPs have made them a highly interesting material class for a broad range of biomedical applications, including enzyme mimetics, the so-called ‘nanozymes’ [40].

In the field of bioimaging, gold nanostructures have been the subject of intensive research owing to their exceptional optical and plasmonic properties, as well as high chemical stability. The occurrence of size- and shape-dependent surface plasmon resonance (SPR) has led to the fabrication of gold NPs in various sizes and shapes, including rod-shaped, star-shaped, and nanoshells [41]. Aside from imaging applications, the specific absorption of light followed by the conversion into heat energy has made this particle type particularly interesting for heat ablation of cancer cells, a process that is called photothermal therapy (PTT), and allows for their simultaneous use in diagnostics and therapy [42]. Other near infrared-emitting structures, such as carbon nanotubes or lanthanide-doped NPs, are likewise useful for bioimaging and PTT applications; however, their potential toxicity has so far limited their employment in clinical trials [43].

### 3.3. Composite NPs

The combination of several materials in one multi-component particle, such as core-shell or hybrid structures, allows for the fabrication of nanoplatforms that overcome the limitations of single-component structures. In fact, composites often possess superior solubility and biocompatibility compared to individual materials [44]. While silica coatings prevail in the field of inorganic-inorganic composites [45,46], combinations of, e.g., silicon nanostructures modified with metal or metal oxide nanoparticles have recently demonstrated highly interesting properties for biosensing applications [47]. Moreover, many multi-component particles combine the advantageous properties of organic and inorganic structures, e.g., using polymer-encapsulated magnetic particles [48] or metallic nanoparticles with antimicrobial activity and biodegradable polysugar-shells [49]. Such core-shell structures are beneficial to prevent leaching of potentially toxic core-ions while providing a chemically active surface for the decoration with biomolecules. Formation of hybrid nano-colloids can either be performed as post-synthetic encapsulation step after core NP formation or in situ during NP synthesis [50]. Alternatively, to spherical nanoparticles, multi-compositions comprised of two-dimensional structures, such as graphene oxide, and inorganic or organic nanoparticles have been developed based on their unique theranostic performance [51].

## 4. Conjugation Mechanisms of CPP to NP Surfaces

**Figure 2:**
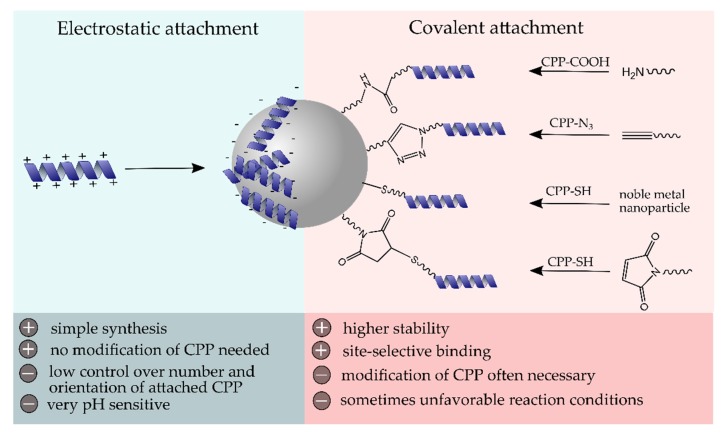
Schematic surface modification of NPs with cell-penetrating peptide (CPP) using electrostatic or covalent coupling strategies and their major advantages and disadvantages.

As soon as NPs enter a biological system, their laboratory identity transforms into a biological identity. In fact, their fate is highly influenced by their physicochemical properties, such as particle size and surface modification, that determine the biodistribution, blood circulation time, protein corona formation, toxicity, and immunological response [52]. Therefore, the post-modification of NPs with biocompatible and functional (bio)molecules is commonly performed. Generally, the cell membrane represents a biological barrier that functional molecules or particles have to overcome to take full effect inside the cell. The effective internalization of CPPs has made them very useful tools for the transport of various cargos, including NPs, across cell membranes [4]. Since the first attempt of producing CPP-NP conjugates as described by the group of Weissleder et al. in 1999, who reported a 100-fold higher internalization into lymphocytes compared to nonmodified particles [53], numerous efforts have been made in the formation of conjugates of inorganic, as well as organic and hybrid nanostructures, with CPPs. The attachment of CPPs to NP surfaces can generally be performed via electrostatic interactions or through covalent coupling strategies (Figure 2). In this chapter, we have intended to highlight some recent conjugation strategies and discuss the advantages and disadvantages of the conjugation mechanisms.

### 4.1. Non-Covalent Attachment

Self-assembly of materials and biomolecules with opposite surface charges represents the simplest modification strategy for the surface decoration of NPs. However, this electrostatic interaction is highly dependent on the ionic strength and pH value and offers, compared to covalent surface linkages, less control over the number of attached molecules, as well as their orientation [54]. Moreover, a ligand exchange in the presence of other highly charged biomolecules is likely to occur and should be investigated to determine the conjugate stability in a biological environment. In some cases, the slow disintegration of bio-conjugates is advantageous, e.g., when it comes to a pH-dependent removal of biomolecule layers that trigger the release of drug molecules from the core material. To date, the often strong positive net charge of CPPs has been exploited for their self-assembly on the surface of negatively charged nanostructures. For instance, Tan and co-workers prepared mesoporous silica particles with strong negative zeta potential (−32 mV) and large pores up to 11 nm, which they used for the noncovalent loading with penetratin [55]. Lower loading rates of CPPs were observed after precedent surface modifications of silica particles with polyethylene glycol (PEG) (average molecular weight 4000 and 10,000, respectively), presumably related to the increase in zeta potential, as well as shielding effects of long PEG chains.

Moreover, our group has recently investigated the internalization efficiency of CPP-NP conjugates based on the electrostatic interactions of silica particles and sC18 [56]. The presence of deprotonated hydroxyl functionalities on the surface of silica particles at neutral pH allowed for simple self-assembly of positively charged CPPs on the NP surface, which resulted in strongly positively charged CPP-silica conjugates. Although conjugates were noncovalently linked, they exhibited high stability in an aqueous environment, even in the presence of serum proteins, indicating a strong CPP-silica interaction. As-prepared nanoconjugates represented excellent internalization efficiencies that were even superior to the individual components.

The internalization efficiency of different CPPs was recently investigated by Cai et al., who noncovalently attached four different CPP, namely, low molecular weight protamine (LMWP), penetratin, Tat, and R8, to the negatively charged surface of PLGA NPs through electrostatic forces and investigated their use for cochlear drug delivery applications [57]. In direct comparison with other CPPs, LMWP provided enhanced cochlear delivery of NPs in vivo, based on its strong positive net charge, as further discussed in Section 5.1.

Instead of performing post-synthetic surface modifications of NPs with CPPs, in situ complexation approaches with negatively charged molecules can be employed for the formation of polyelectrolyte complex NPs. For instance, He and co-workers described the successful synthesis of insulin-penetratin nanocomplexes through electrostatic forces, resulting in homogenous spherical NPs with a mean size of 75 nm [58]. Upon agitation of as-prepared particles at 37 °C in phosphate-buffered saline (PBS), the release of insulin could be measured over several hours, indicating the disintegration of nanocomplexes, a process that could be retarded though surface coverage of NPs with hyaluronic acid (HA), which could gradually dissociate from the NP core upon pH change. This surface coating is especially useful for the oral administration of NPs, as HA provides significant protection to the encapsulated insulin in the gastrointestinal tract while reducing interactions of the NPs with the mucin network.

### 4.2. Covalent Attachment

Covalent linkages of CPPs to NPs represent the most prominent modification strategy for both organic and inorganic NP types as they provide high stability and precise control over site-selectivity, key requisites to preserve function and properties of the peptides. In order to avoid steric hindrance on the particle surface, spacer molecules, such as PEG, are, in many cases, additionally employed. One of the most frequently used covalent conjugation mechanisms is the carbodiimide-based coupling strategy. It relies on the amide bond formation between free amines and carboxylic acid groups and is commonly performed in an aqueous environment in the presence of carbodiimides, such as 1-ethyl-3-(3-dimethylaminopropyl)carbodiimide (EDC). Being comprised of amino acids as basic building blocks, biomolecules, such as CPPs, offer amino, as well as carboxylic acid groups, making carbodiimide reactions a versatile method with high practicability and synthetic accessibility. For instance, He et al. recently described the synthesis of Yb^3+^ and Er^3+^ doped NaYF_4_, upconverting NPs that were subsequently loaded with siRNA and a photosensitizer into their onion-like structure. Following a surface modification with polyethylenimine (PEI), which provided numerous free amino groups, R8 was covalently linked via carbodiimide coupling [59]. Upon irradiation with 808 nm, visible emission was generated that led to the activation of the photosensitizer and resulted in reactive oxygen species (ROS) formation, which favored the disintegration of NPs and thus the release of siRNA. Surface decoration with R8 assisted the cell internalization and endosomal escape of NPs, demonstrating significantly higher uptake rates compared to control NPs without the CPP.

While the abundant presence of amino and carboxylic acid groups is very beneficial for the covalent attachment of CPPs since no additional pre-modification steps are required, the high number can also lead to the unspecific binding that can alter the molecular functionality. Therefore, many approaches for the covalent linkage of CPPs to NPs make use of the less frequent functionalities, such as thiols, that are present in cysteine. Free thiols can interact with maleimide residues to form a stable carbon-sulfur bond, as demonstrated by various groups for the covalent attachment of CPPs, such as RF, LMWP, or Tat to liposomes, dendrimers, or protein-based NPs [60,61,62]. Alternatively, the high affinity of noble metals to sulfur, which derives from the soft acid–soft base interaction, can be employed for the direct linkage of sulfhydryl-containing CPPs to the surface of metal NPs, as recently demonstrated for R8 and Tat conjugation to silver, gold, and palladium NPs [63,64,65,66,67].

Although Michael-type additions, such as maleimide-thiol reactions, are part of the family of click reactions, which stand out based on their high selectivity, stereospecificity, yield, as well as mild reaction conditions, formed succinimide-thioethers are prone to exchange reactions with other thiols or can undergo retro-Michael additions under physiological conditions [68]. Therefore, alternative click reactions, such as copper-catalyzed azide-alkyne cycloadditions, that selectively deliver 1,4-disubstituted 1,2,3-triazoles have been used as a very selective and stable conjugation strategy. For instance, Perillo et al., as well as Han et al., reported on the covalent linkage of Tat and gH625 onto the surface of polypeptide micelles and PEG-coated liposomes, respectively, using the copper-catalyzed click chemistry [69,70]. Nevertheless, the potential toxicity of copper ions currently leads the way towards copper-free click alternatives, e.g., based on cyclooctyne derivatives [71].

Table 2 summarizes some recent approaches on the CPP conjugation to organic or inorganic NPs and highlights the employed linkage strategy.

## 5. Physicochemical Properties of CPP-NP Conjugates

Combining the beneficial core properties of NPs with biocompatible and cell interactive biomolecules on the vehicle surface allows for their implementation in various fields of biomedicine, including bioimaging, targeting, and therapeutic applications. Nevertheless, the distinct properties of CPPs and NPs can be altered upon conjugation, whether covalent or non-covalent, thereby significantly changing the cellular interactions. Therefore, in-depth physicochemical analytical methods are needed to gain further insight into this complex interplay. Table 3 summarizes some of the most employed characterization techniques to investigate the properties of CPP-NP conjugates.

Aside from physical, chemical, and biological characterization techniques, computational quantum mechanical modeling, e.g., based on density functional theory (DFT), has demonstrated enormous potential for elucidating the chemical bond formation between NP surface and the peptides.

In this chapter, we aimed to highlight some properties that are dependent on the CPP or the carrier and have a significant effect on the CPP-NP conjugate formation and discuss the uptake and intracellular fate of conjugates in comparison to their individual components.

### 5.1. Influence of Charge on CPP-NP Conjugate Formation

Given the negative charge of cell membranes at physiological pH, which is based on the low acid dissociation constant (pKa) of phosphate groups of phospholipids, the surface charge of CPP-NP conjugates plays a pivotal role for their cellular interactions [72,73]. The usually high positive net-charge of CPPs is very beneficial for their self-assembly on the surface of negatively charged NPs, although the interactions between CPPs and NPs at the molecular level are not completely understood. Grasso and co-workers shed new light into this field using molecular dynamics to reveal the functional groups of peptides responsible for electrostatic interactions with silica NPs, comparing six differently charged CPPs [74]. Their results suggested that the silica protonation state strongly affected the CPP binding affinity and that mainly charged residues, such as arginine and lysine, were responsible for the strong interactions based on hydrogen bonds. In the case of amphipathic CPPs, such as MAP, the N-terminal tail plays a significant role for the NP adsorption. Therefore, slight changes in peptide sequences that alter their net charge have a significant influence on their binding affinity and can, for example, be used to control the density of CPPs on negatively charged NP surfaces [56]. Moreover, the number and order of amino acids in the peptide sequence, especially charged ones, have a significant effect on their internalization efficiency, also when conjugated to NPs. In direct comparison to other CPP-PLGA conjugates, LMWP-coated PLGA NPs have been internalized at the highest rates, presumably due to the high arginine content, which, under physiological pH, is able to form hydrogen bonds with the negatively charged phosphates and sulfates on the cell surface membrane [57,75]. Additionally, as previously mentioned, the guanidine groups present in arginine have been shown to assist the endosomal escape of NPs due to the proton sponge effect, which is especially useful for drug delivery applications [59].

### 5.2. Influence of Carrier on CPP-NP Conjugate Formation

Generally, the attachment of CPPs to the NP surface has been shown to be beneficial for the accessibility of CPPs, which potentially enhances recognition of the peptide with the cell surface [76]. Nevertheless, the chemical interaction between functional groups of the peptide and those of the NPs can have a significant influence on the formation of the secondary structure. CD spectroscopy studies of sC18-silica conjugates have demonstrated the formation of a less pronounced alpha-helical structure compared to the free CPPs, even though a noncovalent approach has been used for the conjugate formation [56]. The multiple hydrogen bond formation between cationic amino acids and deprotonated surface silanol groups presumably has led to reduced flexibility of the CPP backbone upon conjugation, which impairs the full development of an alpha helix. Interestingly, as-received conjugates have still demonstrated much higher internalization efficiencies compared to the free peptides and the free NPs, indicating that the charge rather than the secondary structure of CPP constitutes the main driving force for membrane translocation.

Moreover, there are several studies available that demonstrate that the carrier size plays a pivotal role when it comes to crossing cell membranes and their fate inside the cell [77]. This relationship has also been evidenced in several studies on CPP-NP conjugates that compared carriers of different sizes. For instance, Oh et al. synthesized Au NPs in sizes ranging from 2.4 to 89 nm and covalently conjugated CPPs via a PEG linker [78]. After incubation of COS-1 cells with as-prepared CPP-NP conjugates, significant differences in cellular uptake and final intracellular localization were observed based on the carrier size. While the smallest 2.4 nm NPs translocated into the cell nucleus, intermediate 5–8 nm-sized NPs were found in the cytoplasm, and 16 nm and larger samples did not enter the cells but were localized in the cell periphery. Guarnieri and co-workers observed that differently sized (2.5, 5, and 20 nm) platinum NPs that were functionalized with a membranotropic peptide, namely, gH625, were internalized into cells at much higher rates compared to non-functionalized NPs [79]. However, only in case of 2.5 nm NPs, the functionality of the CPP was retained, enabling cytosolic delivery of Pt NPs via diffusion across the cell membrane.

### 5.3. Influence of CPP-NP Conjugate Formation on Toxicity, Biocompatibility, and Stability

While positively charged NPs have been shown to have stronger cell membrane interactions and higher cell uptake rates compared to neutral or negatively charged counterparts, they may disrupt the integrity of the cell membrane and, therefore, lead to higher cytotoxicity [80,81,82]. On the other hand, cation-rich CPPs that possess a high positive net charge have demonstrated low toxicity even at concentrations of 100 µM, although the attachment of cargo has shown to have a significant influence on the cytotoxicity for some CPPs [83]. Nevertheless, in most reported cases, no increase in cytotoxicity was observed when CPPs were combined with NPs [56,78,84,85]. Only a few examples, for instance, by Liu and co-workers, demonstrated that the concentration of attached CPP, in this case, R9, which was linked to the fusogenic peptide INF7, to CdSe/ZnS quantum dots (QDs) dramatically changed the observed cytotoxicity on A549 cells [86].

Generally, many inorganic NPs suffer from toxicological issues, related to the slow degradation under physiological conditions, which results in the release of often harmful metal-ions. Therefore, coatings that rely on peptides have been shown to stabilize NPs, thereby reducing their toxicological profile, and additionally be helpful in directing carriers to the place of interest as well as to support their clearance [87]. Moreover, Bajaj and co-workers recently reported on peptide functionalized Au or Ag NPs, which demonstrated enhanced stability compared to their non-functionalized counterparts, with covalent modification strategies being superior over electrostatic interactions in preventing NP agglomeration [88].

## 6. Application of CPP-NP Conjugates in Cancer Medicine

According to the World Health Organization (WHO), cancer is the second leading cause of death worldwide (www.who.int), and, therefore, novel treatment strategies, including improved anticancer drugs, are highly appreciated. Since many chemotherapeutic drugs suffer from poor pharmacokinetics, exhibiting fast clearance and limited accumulation at the target site, combination with NPs may offer a considerable benefit. Notably, by virtue of their small size, some NPs are also able to cross the blood-brain barrier and, thus, offer opportunities for the diagnosis and treatment of difficult to reach targets, such as brain tumors [89]. Furthermore, multimodal systems can be generated within one NP as a platform. For instance, drugs can be encapsulated, and the surface decorated with several functional moieties, like homing devices, CPPs, and, e.g., PEG chains. In the following chapter, we have provided some examples of the last years in which such multifunctional complexes were applied in different aspects of cancer research.

### 6.1. Cancer Targeting

One of the main disadvantages of anticancer drugs is their low target selectivity, promoting their exposure to healthy tissue, which finally leads to severe side effects. Therefore, it is of utmost importance to address this issue and to combine chemotherapeutic drugs with cancer-targeting groups. Since CPPs are usually nonselective and principally able to enter all kinds of cells, CPP-NP systems for cancer diagnosis or therapy are usually combined with a targeting moiety, in most cases, biomolecules that recognize surface or membrane receptors of cancer cells. In addition to this, adding selective affinity labels may enable traversing the blood-brain barrier (BBB), as recently demonstrated by dos Santos Rodrigues et al., who generated lipid-based NPs and decorated them covalently with a CPP and a recognition motif for transferrin receptors (Tf) overexpressed on the BBB. Their results indicated the superior ability of Tf and Tat-modified liposomes to cross the barrier layer and to transfect neuronal cells in an in vivo set-up, underpinning the high value of this novel brain-targeted gene delivery system [90].

In another approach, specific cancer-targeting peptides are used to exploit the unique characteristics of the tumor environment or cancer cells. In this respect, tumor homing peptides that specifically bind to receptors overexpressed on cancer cells are established, either by using phage display techniques or by rational design and modification of the natural peptide ligands. For example, the short peptide motif Arg-Gly-Asp (RGD) is specifically recognized by several α*_v_* integrin receptors frequently overexpressed on cancer cells [91]. RGD and variants thereof are used in many drug applications to generate peptide–drug conjugates but serve also in many other treatment strategies as tumor-targeting sequences [92,93]. Several examples of using the RGD sequence to direct NP cargos to cells have been already described in the literature [1]. Recently, one interesting report was presented by Kostiv et al., who designed luminescent lanthanide-based NPs (NaYF_4_:Yb^3+^/Er^3+^), which they decorated either with a cell adhesive RGD-containing peptide or a Tat-derived CPP [94]. The peptides were in both cases covalently attached via copper-catalyzed alkyne-azide click reaction. Notably, depending on the used peptide, the authors observed either localization at the membrane of HeLa cells owing to specific binding of the peptide to the corresponding integrins; or, in case the CPP was attached, they found an intracellular accumulation of the peptide-NPs. This underpins how the choice of the attached peptide may specify the future application of the NP construct: the herein reported probes could be either useful tools for targeted in vivo cell imaging or suitable for photodynamic therapy, for which deep penetration into the tissue is favorable.

To achieve an effective tumor targeting, it is crucial to know the expression and accessibility of the defined receptors in the target cells. Willmore et al. recently generated barcoded silver NPs as a tool for auditing affinity ligand receptors in cells [95]. They used two different tumor penetrating peptides as affinity ligands, which were coupled via streptavidin/biotin binding to the particles. Receptor-dependent binding and uptake of the so-modified NPs by either PPC-1 or M21 cells were demonstrated. More interestingly, by using isotopic multiplexing and ICP-MS-based phenotyping of silver nanoparticles (AgNPs), preferential and selective binding, as well as internalization depending on the targeting peptides, was further underlined. This strategy indeed holds great promise as a ratiometric system for homing peptide specificity and validation studies and is potentially useful for in vivo tumor profiling of peptide-NP conjugates.

On the other side, so-called cancer-targeting or anticancer peptides (ACPs) have been reported [96], which are, owing to their specific nature, attracted by the specific phenotypes of cancer cells and their surroundings. In fact, cancer cells differ from healthy cells since they display more negatively charged cell surface components, including also negatively charged headgroups of the lipid bilayer forming phospholipids. This promotes the interaction with the cationic charged ACPs and is partly responsible for the relative tumor cell selectivity. However, many other reasons count for the design of such cancer-targeting sequences as the acidic extracellular environment or tumor microenvironment hypoxia [97]. Several successful combinations of drug-loaded NPs with cancer-targeting peptides have already been demonstrated [98]. In one recent study, the cellular uptake of gold nanorods and gold NPs, respectively, modified with the anticancer peptide SVS-1 was analyzed [99]. For functionalization with the peptides, the particles were first coated with a polymer shell having reactive amine groups that were further modified with maleimide residues and then coupled to the N-terminal cysteine of the SVS-1 peptides. Interestingly, uptake in HeLa cells revealed the cytosolic distribution of both NP species, indicating that endocytosis is probably not the dominant entry mechanism. Given the fact that the materials displayed no cytotoxicity, this approach might offer a direct and more effective strategy for the intracellular and cytosolic delivery of nanoconjugates.

Notably, also CPPs may have an intrinsic cancer selectivity that is assumedly based on present cationic amino acid residues within their sequences. Moreover, CPPs may be rationally modulated to target cancer cells [100]. Recently, Carnevale et al. compared the uptake profiles of several different CPP-NPs in cancer cells versus cancer-resistant cells [76]. In fact, the authors used eight different CPPs, which were attached via bidentate coordination using the thiol and amine of cysteine to the NP surface to generate multi-shelled QDs, and tested their ability to enter cancer cells. Interestingly, besides peptide-specific uptake preferences, the authors observed unique QD-CPP fluorescence profiles dependent on the individual cell types investigated. This effect was probably due to the differential phenotypic expression of cell membrane surface components between various tissue types, as well as on the drug resistance status. This study gives important insights into the use of CPPs in cancer cell lines since variable internalization efficiencies dependent on the CPP used were observed.

Summarizing, for selective tissue-targeting the sole use of a CPP is often not sufficient, and, thus, the additional decoration of NPs with homing peptides or direct modification with lytic anticancer peptides may be favorable. Importantly, for all approaches reported, the decoration of the NPs with CPPs and/or targeting peptides has led to an overall increased cellular uptake, highlighting the beneficial combination of both parts.

### 6.2. Cancer Imaging

Owing to their variable physicochemical properties and structures, NPs can be tailored for high-efficiency diagnostic tools and represent attractive possibilities for tumor diagnosis. Moreover, based on their characteristics and ease of modification with functional groups, NPs can be used as multifunctional platforms for imaging purposes, involving optical imaging, radionuclide imaging, magnetic resonance imaging, or ultrasound imaging. Often, such properties are combined, as recently reported by Perillo et al., who designed multifunctional NP systems composed of superparamagnetic iron oxide NPs that were additionally labeled with a fluorescent dye. Subsequent coating of the surface with a CPP facilitated cell entry in human mammary carcinoma MDA-MB-231 cells [101]. Since the peptide was enriched with an additional cysteine, it could be easily introduced within the PEG shell in a certain orientation- and sequence-specific coupling strategy, being indeed indispensable for the expected biological response. Thus, intracellular accumulation of these multifunctional NP systems only succeeded after chemical coating with the CPPs.

Such multifunctional approaches have been also used by Liang et al. to generate nanocomposites useful for targeted imaging and selective killing of cancer cells [102]. Herein, lanthanide NPs were coated with a silica layer encapsulating a photosensitizer, and additionally bioconjugated to monoclonal antibodies targeting epithelial cell adhesion molecules (EpCAM) on human colon carcinoma HT-29 cells. Upon selective binding to the cancer cells and radiation, the energy was transferred from the NP to the photosensitizer releasing reactive singlet oxygen responsible for selective killing of the cells. Another such theranostic application based on the use of CPP-coated silica NPs was described in the work of Yu et al. [103]. As already mentioned, silica NPs stand out by their advantageous properties, such as high surface area, uniform pore size, high loading capacity, non-toxicity, and ease of functionalization. In this work, the mesoporous silica NPs were coated with CPPs and a fluorogenic apoptosis-detecting peptide [104]. Again, only with the help of the CPP, high cellular uptake in HeLa cells was achieved. When loading the system additionally with apoptosis-inducing cargos, the efficient drug release and biological effects were detectable by the self-reporting attached peptide using two-photon fluorescence microscopy. Owing to several key advantages, such as no visible endosomal entrapment and direct biological read-out, these platforms may be further developed for future applications in personalized medicine.

### 6.3. Cancer Therapy

The application of CPP-modified NP drug carriers attracts great interest in developing novel promising cancer treatments. Interestingly, by combining different composites, limitations that come with CPPs or inorganic particles may be circumvented. For instance, as is with all peptide therapeutics, also CPPs suffer from low proteolytic stability hindering, e.g., oral administration and affecting overall bioavailability. One way to improve CPP properties was recently illustrated when iron oxide NPs were non-covalently modified with CPP-conjugated chitosan. The chitosan modification greatly improved the performance of the drug loading capacity, in this case, siRNA, as well as the overall biocompatibility and bioavailability of the system [105]. Additionally, this cationic chitosan used compacted, not only the nucleic acid payload but also contributed to improved stability of the CPPs. Particularly for cargos, such as therapeutic nucleic acids, which are prone to fast degradation and for which membrane translocation is hardly restricted, peptide-NP compositions might be suitable transfection vectors. In this respect, Panigrahi et al. presented a novel class of short tryptophan-containing CPPs, which were employed for the formation and simultaneous coating of gold NPs. Beside physicochemical characterization, those constructs were also investigated concerning their interplay with siRNA. For this, the authors used fluorescently labeled siRNA and proved its high intracellular accumulation in human colon cancer cells (HCT 116). More interesting was that they demonstrated successful gene silencing with transfection efficiencies comparable to commercially available Lipofectamine 2000 [106]. Notably, this is one of the rare studies in which successful drug delivery was obtained even in the presence of serum, proving again the favorable cooperation of CPPs and, in this case, anisotropic gold NPs.

Previously, silver (Ag) NPs have been reported to exhibit anticancer effects. To guarantee enhanced cellular uptake, AgNPs were functionalized with different CPP variants, and accumulation in breast cancer MCF-7 cells was proven [107]. In this work, the NPs were covalently modified with cationic CPPs via amide bond formation, and successful coupling was proven by different techniques, like UV spectroscopy and zeta potential measurements. With the latter method, a decrease in the negative potential of AgNPs after CPP modification was demonstrated. Consequently, the reduction of the electrostatic barrier was induced by the CPP layer, finally leading to the observed improved cellular uptake and higher cytotoxicity of these constructs.

Another main application in which NPs are used in the field of cancer therapy is small molecule drug delivery. The intention is to safely release the chemotherapeutic with high efficiency at the target site. Also, in this case, the additional functionalization with CPPs is thought to support increased tissue and cell penetration, as, for instance, the already noted BBB penetration. Thus, Morshed et al. studied CPP-conjugated gold NPs as delivery vehicles for doxorubicin to be shuttled to brain metastatic breast cancer cells [108]. Besides their in vitro characterization, the conjugates were investigated in vivo, where they indicated promising accumulation and selective cytotoxicity within invasive intracranial MDA-MB-231-Br tumors in a proof-of-principle xenograft mouse model, suggesting a promising role for treating brain metastatic breast cancer lesions. Hence, for future studies, those Tat-dox-AuNPs may be favorable composites that are able to induce apoptosis and, thus, significantly contribute to prolonged survival in tumor-bearing mice. In another study, such composites were further improved by the additional introduction of a targeting moiety. Therefore, biohybrid gold NPs were modified with the CPP Tat and cancer-targeting antibody to increase target site delivery of included doxorubicin. The authors were also interested in the intracellular release of doxorubicin from these hybrid NPs, which they monitored by using surface-enhanced Raman spectroscopy (SERS). In fact, in situ monitoring by SERS showed the efficient release of the drug inside the cells, and its potent anticancer effect was validated by cell viability assays, demonstrating the versatility of this strategy [109]. Another small molecule drug out of the group of tyrosine kinase inhibitors, namely, afatinib, was used in the study of Hong et al. to inhibit HER2 and HER3 phosphorylation since this is related to tumor resistance, metastasis, and invasion, resulting in poor outcome of anti-colorectal cancer therapy [110]. Herein, a polymeric NP formulation for drug delivery was prepared, in which NPs were surrounded by lipids modified with a targeting ligand and a pH-sensitive CPP. These constructs were evaluated concerning their biological effect on colorectal adenocarcinoma Caco-2 cells. The results proved a remarkable induction of apoptosis and inhibition of resistance of Caco-2 cells, proposing that those multifunctional NPs might pave the way to increased sensitivities of colon cancer cells to afatinib.

Within all these examples of CPP-NP conjugates, the role of the CPP was to achieve increased target site accumulation, as well as to improve overall cellular uptake. Remarkably, independent of the CPP attached, even when novel CPP sequences were used as, for instance, in the study of Panigrahi et al. [106], enhanced membrane translocations were observed for NPs. Additionally, it is apparent that, in most cases, the applied CPPs were of cationic nature, a property that is probably key to lower the negative potential of certain NPs and, thus, to improve membrane surface interaction that is important for a successful uptake process (see chapter 2.2). On the other side, it is also indisputably shown that NPs serve as advantageous platforms to enhance CPP stability. When at the same time drugs are incorporated within these systems, they are safely transported and released at their target side.

## 7. Current State-of-the-Art and Future Perspectives

Given their nanoscopic dimension that falls within the same size regime as many intracellular structures, NPs are well suited as versatile tools in biomedicine. Especially inorganic NPs that offer inherent optical, magnetic, or electric features while providing defined surface chemistry suitable for the attachment of multifunctional biological molecules are of interest for combined therapeutic and diagnostic applications. NPs have been shown to improve the pharmacokinetics of biomolecular drugs by protecting them from fast blood clearance or rapid enzymatic degradation, ensuring safe delivery to the site of action. When combined with CPPs, permeation into target cells is facilitated, leading to high intracellular accumulations of the carrier. During the last years, tremendous efforts have been made to design and modulate novel CPP sequences, exhibiting increased membrane permeation abilities and improved target specificity. In addition, identifying shorter sequences is of great value when taking easier synthesis pathways and lower production costs into account. Importantly, computational methods attract more and more notice to predict novel CPP sequences [111].

However, gaining insight into the complex interactions between CPPs and NPs and identifying suitable coupling mechanisms that provide sufficient stability and biocompatibility without impairing the peptide functionality are key challenges toward their clinical application. Although much efforts have been made to develop smart multimodal signal-generating vectors, the increasing complexity of prepared nanostructures at the same time represents ambitious and often laborious syntheses and thus a major challenge with regard to reproducible synthetic protocols. Therefore, there is a great demand for simple ‘one-pot’ synthetic routes that deliver functional nanoplatforms, which can be combined with a novel and smart linker techniques to provide colloidal stability under physiological conditions.

Additionally, even though NPs have found their way into clinics and CPP-based clinical trials are tremendously increasing, no FDA approved CPP-NP conjugate is currently available [112]. One major obstacle is the lack of long-term toxicity studies for many nanomaterials, including particles made of lanthanides that represent a highly interesting class of optical NPs. Moreover, the poor selectivity of many treatments can only be overcome by attaching both tumor-homing devices as well as CPPs to increase targeting of those constructs to the tumor tissue and to facilitate tumor penetration. Owing to the possibilities of parallel modifications, the multi-functionalization of particles with both moieties is feasible.

Interestingly, a lot of research has been invested in the development of strategies for oral administration since this is meant as the ideal mode of drug administration [113]. In this respect, Tan et al. recently presented CPP-PEG-modified mesostructured silica NPs that greatly enhanced the bioavailability of therapeutic peptides and proteins. Significantly promoted mucous permeation and oral availability of drug payloads were observed and demonstrated the potential of their study for future developments in this direction [55].

Substantial efforts in the design of CPP-NP platforms, adapting the basic principles for medical application, have led to significant progress in nanomedicine and allow for the transport of diagnostic and/or therapeutic vehicles across almost insurmountable barriers, such as the BBB. The next challenge is to find suitable engineering techniques to tightly control size and morphology, to improve specificity and selectivity, and to understand structural and environmental factors important for their activity under physiological conditions.

## Figures and Tables

**Table 1:** List of some representative cell-penetrating peptides (CPPs) that have been used to create CPP-NP (nanoparticle) constructs and that are discussed within this review.

Name	Sequence	Ref.
Tat (48–60)	GRKKRRQRRRPPQ	[5,6]
Penetratin	RQIKIWFQNRRMKWKK	[14]
VP22	RPRAPARSASRPRRPVE	[13]
R9	RRRRRRRRR	[10]
sC18	GLRKRLRKFRNKIKEK	[12]
MAP	KLALKLALKALKAALKLA	[15]
Pept1	PLILLRLLRGQF	[16]

**Table 2:** Overview of NP-CPP conjugates and employed conjugation mechanism.

Nanocarrier	CPP Type	Conjugation Mechanism	Ref.
**Inorganic**			
Silica NPs	penetratin	electrostatic	[55]
Silica NPs	sC18 (and derivatives)	electrostatic	[56]
NaYF_4_:Yb, Er	R8	carbodiimide coupling	[59]
Pd-nanosheets	Tat	Pd−S coupling	[63]
Au NPs	R8	Au–S bond	[64]
Au NPs	Tat	Au–S bond	[65]
Au NPs	Tat	electrostatic / Au–S bond	[66]
Ag–Fe_3_O_4_ NPs	Tat	Ag–S bond	[67]
**Organic**			
PLGA NPs	Tat, LMWP, penetratin, R8	electrostatic	[57]
Insulin NPs	penetratin	electrostatic	[58]
liposomes and solid lipid NPs (SLNs)	RF	Malemide-thiol coupling	[60]
Albumin NPs	LMWP	Malemide-thiol coupling	[61]
PAMAM dendrimer	Tat	Malemide-thiol coupling	[62]
PEG-coated liposomes	gH625	Cu catalyzed azide–alkyne cycloaddition	[69]
Polypeptide micelles	Tat	Cu catalyzed azide–alkyne cycloaddition	[70]

**Table 3:** Overview of commonly used analytical methods to determine the physicochemical properties of NP-CPP conjugates.

Investigated Parameter (s)	Analytical Method (s)
CPP secondary structure	Circular dichroism (CD) spectroscopy, infrared spectroscopy
Quantitative cellular uptake	Flow cytometry, mass spectrometry
Qualitative cellular uptake	Confocal microscopy
Surface charge	Zeta potential
NP size and morphology; in some cases, also CPP shell thickness	Transmission and scanning electron microscopy, atomic force microscopy
CPP quantification	High-performance liquid chromatography (HPLC), Bradford assay, UV-vis spectroscopy
CPP binding affinity and surface coverage	Isothermal calorimetry (ITC), differential scanning calorimetry (DSC), SPR
Hydrodynamic size	Dynamic light scattering (DLS)
Functional surface groups and CPP-NP binding	X-ray photoelectron spectroscopy (XPS)

## Abbreviations

ACP	Anticancer peptide
AMP	Antimicrobial peptide
BBB	Blood-brain barrier
CD	Circular dichroism
CPP	Cell-penetrating peptide
DFT	Density functional theory
DLS	Dynamic light scattering
DSC	Differential scanning calorimetry
EDC	Ethyl-3-(3-dimethylaminopropyl)carbodiimide
EpCAM	Epithelial cell adhesion molecule
FDA	Food and Drug Administration
HIV	Human deficiency virus
HA	Hyaluronic acid
HPLC	High performance liquid chromatography
ICP-MS	Inductively coupled plasma mass spectrometry
ITC	Isothermal calorimetry
LMWP	Low molecular weight protamine
LD	Linear dichroism
MAP	Model amphipathic peptide
NP	Nanoparticle
PBS	Phosphate buffered saline
pDNA	Plasmid deoxyribonucleic acid
PEG	Polyethylene glycol
PEI	Polyethylenimine
pKa	Acid dissociation constant
PLA	Poly(lactic acid)
PLGA	Poly(lactic-*co*-glycolic) acid
PMMA	Poly(methyl methacrylate)
PNA	Peptide nucleic acid
PTD	Protein transduction domain
PTT	Photothermal therapy
QD	Quantum dot
ROS	Reactive oxygen species
RGD	Arginylglycylaspartic acid; Arg-Gly-Asp
SERS	Surface-enhanced Raman spectroscopy
siRNA	Small interfering ribonucleic acid
SLN	Solid lipid nanoparticle
SPR	Surface plasmon resonance
Tat	Transactivator protein
Tf	Transferrin
TLA	Three-letter acronym
WHO	World Health Organization
XPS	X-ray photoelectron spectroscopy
